# The History of Alcoholic Liver Disease: From an Unrecognized Disease to One of the Most Frequent Diseases in Hepatology

**DOI:** 10.3390/jcm10040858

**Published:** 2021-02-19

**Authors:** Helmut K. Seitz, Manuela G. Neuman

**Affiliations:** 1 Centre of Liver and Alcohol Diseases, Ethianum Clinic, 69115 Heidelberg, Germany; 2 Faculty of Medicine, University of Heidelberg, 69117 Heidelberg, Germany; 3 In Vitro Drug Safety and Biotechnology and the Department of Pharmacology and Toxicology, Temerty Faculty of Medicine, University of Toronto, Toronto, ON M5G 1L5, Canada; manuela.neuman@utoronto.ca

**Keywords:** alcoholic liver disease, chronic alcohol consumption, hepatotoxicity, liver biopsy, liver inflammation

## Abstract

This review describes the history of alcoholic liver disease from the beginning of the 1950s until now. It details how the hepatotoxicity of alcohol was discovered by epidemiology and basic research primarily by using new feeding techniques in rodents and primates. The article also recognizes the pioneering work of scientists who contributed to the understanding of the pathophysiology of alcoholic liver disease. In addition, clinical aspects, such as the development of diagnostics and treatment options for alcoholic liver disease, are discussed. Up-to-date knowledge of the mechanism of the disease in 2020 is presented.

## 1. Introduction

Chronic alcohol consumption is responsible for more than 200 diseases [1]. Among them, alcoholic liver disease (ALD) is one of the most frequent and most serious complications of chronic alcohol intake. Approximately 50% of all cirrhosis of the liver in Europe is due to alcohol [2]. Perhaps surprisingly, seventy years ago, doctors rejected ALD as a stand-alone disease. Alcohol was not regarded as a toxin for the liver. Indeed, it was none other than Charles Best, the Nobel laureate, who stated in 1949 that alcohol is not more toxic for the liver than a sugar solution [3].

This review article on ALD traces its development from a rarely recognized condition in the nineteen-fifties, to becoming one of the most frequent liver diseases in hepatology today. The purpose of the review is to describe how the puzzle of ALD was put together step-by-step, beginning with pioneering studies in epidemiology, as well as animal studies which demonstrated the toxic role of ethanol. It took a rather long time to understand the many mechanisms in the pathogenesis of ALD and how they work in concert. Even today, many questions remain unanswered. For a long time, no good data existed which showed a relationship between the amount of alcohol intake and the risk for cirrhosis of the liver. It was Werner Lelbach from Germany, together with Georges Pequignot and Albert Tuyns from France, who first demonstrated such a dose–risk relationship. A small number of competing laboratories shaped the possibility of understanding the hepatotoxicity of ethanol and its mode of action. In those early days, the big players in the alcohol field were Charles S. Lieber in New York, Ronald Thurman in Chapel Hill, Yedi Israel in Toronto, and Sam French in Los Angeles, to name only a few. In Japan, Hiromasha Ishii and his team were at the forefront, and in Europe, Christian and Christiane Bode from Germany contributed much to alcohol research. In this review, credit is given to some of the early pioneers in alcohol research. The focus of this review is to illustrate how, step-by-step, major discoveries led to the understanding of the pathogenesis of the disease. Gradually, ALD became recognized by the medical profession, as well as by the public, as one of the most important diseases in hepatology. The article concludes with up-to-date knowledge in the pathogenesis of ALD and summarizes clinical aspects such as prevention, diagnosis and treatment in 2020.

## 2. A Brief History of Alcohol Use and Abuse

Humans have been misusing alcohol for thousands of years. Archeologists have found a grape squeezer in Syria from around 6000 B.C. In the same period, wild grapes grew in the Zagros Mountains, North of the Persian Gulf, between the Tigris and Euphrates Rivers, in the Elemite capital of Susa in Persia (Iran), as well as Uruk and Tello in Mesopotamia (Iraq). The wine arrived in Egypt’s Abydos region by trade. In the tombs of pharaohs there were jars presumably containing wine. The dangers of alcohol abuse are even shown in 3500-year-old hieroglyphs found in Echnaton in Egypt.

Grapes were first cultivated in Jericho, Israel, around 4000 BC. In the Old Testament, it is written that Noah planted grapes following the flood and made wine. (“He, (Noah), drank of the wine and became intoxicated”) (Genesis 9.21–24). Noah was the first known person who cultivated grapes and the first individual described to be intoxicated. On the Arab Peninsula, grapes grew at Bab edh-Dhra, En Besor and Minshat Abu Omar. Wine was traded and shipped to the Ancient Greek and Roman Empires from the Jordan Valley and Nile Delta. The Roman and Greek poets described the pleasures and dangers of drinking wine. In 1530 AC Paracelsus translated the Arabic “al-kuhl” into “alcohol”. In Europe, in ancient times, alcoholic drinks were consumed mainly by the upper classes and the priests.

In Asia, the use of alcohol followed diverse patterns, according to different religious and historical influences at various periods. In China, for example, alcohol has been produced since the Neolithic period. It was used as an anesthetic in traditional Chinese medicine and was a component of alcoholic drinks containing plants such as broomcorn millet, Triticeae grasses, rice, beans, ginger, and yam lily.

In India, records show differences in alcohol use between the Vedic (1500–700 BCE) and post Vedic eras, when Buddhism and Janism (700 BCE to 1100 CE) introduced anti-alcohol doctrines. Post-Vedic developments in the Hindu traditions were also influenced by religion and the caste system. Alcohol consumption was prohibited in the Islamic era (1100–1800 CE). From 1800 to the present, alcohol misuse was observed in the high status caste of warriors/rulers (Kshatriyas). The priests and their scholars (Brahmin caste) still condemn alcohol use.

In Europe and North America, alcohol consumption decreased from the beginning of the twentieth century to the Second World War, only to increase in the 1980s. Over the last 15 years, per capita alcohol utilization slowly decreased in Southern and Western European countries, whereas alcohol consumption increased in Eastern European Countries [1].

In European countries today, only a small percentage of individuals drink far above the normal quantities of alcohol [2,3]. One major exception is Germany, where the per capita pure alcohol consumption in 2017 was approximately 10.9 L with an average of 24 g of pure alcohol per day per person [4]. Alcoholism and alcohol abuse has; therefore, become a major public health problem worldwide.

Harmful alcohol consumption causes approximately 3.3 million deaths per year, moreover it is predisposing alcoholics to tuberculosis [5]. Alcohol-induced liver injury is one of the most significant diseases caused by chronic alcohol consumption. It leads to cirrhosis of the liver and hepatocellular carcinoma (HCC) [2,6].

## 3. Alcoholic Liver Disease (ALD)

### 3.1. Early Studies, Epidemiology, and Risk Factors

Alcohol was believed to be non-toxic based largely on experimental work in rats who were given alcohol in their drinking water. With this technique, ethanol consumption does not usually exceed 10–20% of the total energy intake of the animal. Until the early sixties, the concept prevailed that alcoholic liver disease (ALD) results from malnutrition commonly observed among individuals consuming chronically high amounts of alcohol, rather than being causally related to the use of alcoholic beverages [7,8,9,10]. However, the malnutrition concept became a matter of debate in view of the clinical observation that even humans on a normal diet, without signs of malnutrition, were at risk of ALD. Under metabolic ward conditions with a nutritionally adequate diet, alcoholic fatty liver developed, substantiating that short-term use of alcohol combined with nutritionally adequate diets is responsible for the early manifestation of ALD [7]. These results obtained in humans were subsequently confirmed in experimental animal studies, whereby rats received alcohol in a nutritionally adequate diet [8]. Therefore, and based on the pioneering work of Charles S. Lieber and his associates, the conclusion that alcoholic beverages themselves, rather than malnutrition, causes early stages of ALD was reached [9,10].

Early epidemiologic studies by Werner Lelbach [11], Georges Pequignot and Albert Tuyns [12] in the nineteen-sixties and seventies, clearly demonstrated that a strong and highly significant correlation exists between the daily alcohol intake and the risk of developing cirrhosis of the liver without any threshold.

Meanwhile, hundreds of epidemiologic studies have identified alcohol as an important cause of liver disease worldwide [5,6,13]. In Europe, ALD is still the leading liver disease with more than half a million deaths per year [2], while in the US non-alcoholic fatty liver (NAFLD) disease predominates. The history of ALD is informed by many host factors, such as excessive weight and obesity, female gender, viral co-infection, and iron overload. All these factors are well known to increase the risk of liver damage [14,15].

Although the relationship between alcohol consumption and the risk for cirrhosis of the liver has been undoubtedly demonstrated, it is still surprising that only 10 to 20% of heavy drinkers develop advanced ALD [5,13]. Factors which modify cirrhosis risk include ethnicity, gender, nutritional factors, obesity, and genetics [13].

Among them, the reason why women have a much higher risk of developing ALD compared with men was always a matter of debate [16]. During the last 50 years, a couple of theories have been discussed, such as the difference in body water [17], the lower gastric first pass metabolism of alcohol in women [18], and finally, the role of estrogens [19].

Clinical observations led to the theory that obese individuals have an increased risk for ALD. With the emerging problem of fatty liver and thus NAFLD during the last 20 years, it became clear that obese individuals and patients with other causes of fatty liver indeed have an additional risk of developing ALD when they drink [13,20,21]. Furthermore, their risk to develop HCC is considerably enhanced [22,23].

Alcohol intake also has a deleterious effect on other types of liver disease, including hepatitis B [24] and C [25], hereditary hemochromatosis [26], and, as most recently detected, a1-anti trypsin deficiency [27].

### 3.2. Genetics, a Risk Factor on Its Own

The observation that ALD sometimes occurs within families was not evident at first, nor were the identification of genes which seem responsible for this observation. Evidence has suggested for quite a while that individual susceptibility to the development of ALD after chronic alcohol consumption is influenced by genetic factors, since only 10–20% of heavy drinkers develop cirrhosis of the liver [13]. Clinicians knew that some patients with alcoholic cirrhosis reported a family history of ALD. Furthermore, monozygotic twins have a higher concordance rate for alcohol-related cirrhosis than dizygotic twins [28].

Most recently, the genetic background of ALD has been studied in more detail. Several large, genome-wide association studies demonstrated that patatin-like phospholipase domain-containing protein 3 (*PNPLA3*) and, to a lesser extent, transmembrane 6 superfamily member 2 (*TM6SF2*) as well as membrane-bound *O*-acyltransferase domain-containing protein 7 (*MBOAT7*) are important genetic determinants of risk and severity of ALD [29,30,31,32,33]. *PNPLA3* is closely involved with lipid metabolism and is a risk factor for non-alcoholic fatty liver disease (NAFLD) and hepatocellular cancer (HCC) [34]. The mechanisms by which *PNPLA3* influences the development of ALD are unclear. Mutation in *TM6SF2* can result in hepatic fat accumulation due to a defect in the secretion of very-low-density lipoproteins. Mutation in *MBOAT7* can affect the acetylation of phosphatidylinositol.

Further risk loci have been identified most recently. While the minor A allele in MARC1:rs2643438 decreases the risk for alcoholic cirrhosis, the minor C allele in HNRNPUL1:rs15052 increases the risk [35]. MARC1 protein is involved in the detoxification of various xenobiotics, including acetaldehyde, as well as in the regulation of nitric oxide production, whereas HNRNPUL1, among others, can regulate TGFß, which is a profibrogenic cytokine.

### 3.3. Animal Models (Rodents and Primates)

#### 3.3.1. Lieber-DeCarli Pair Feeding Model

In the early days of alcohol research, alcohol was given to animals in their drinking water. This procedure did not lead to alcohol blood concentrations sufficiently high enough to expect organ damage. In addition, these animals were inadequately nourished. The breakthrough in identifying alcohol as a hepatic toxin was the development of an adequate animal model in which malnutrition, as a cause for the liver disease, could be excluded. It was Charles Lieber who was the first to demonstrate in rodents [36] and baboons [37] that alcohol damages the liver and leads to fatty liver. He also showed how it leads to cirrhosis of the liver in baboons. In this Lieber DeCarli model, rats are pair-fed an isocaloric liquid diet in which 36% of the total calories consisted of alcohol and the control diet contained the same amount of calories as carbohydrates. The content of proteins, lipids, and, most importantly, of total calories, was the same in both diets. The alcohol concentration was approximately 5%, comparable with that in beer. The animals consumed these diets voluntarily. With this alcohol-containing diet, sufficiently high blood alcohol concentrations can be attained. The administration of the alcohol-containing liquid diet results in fatty liver and in the induction of cytochrome P4502E1 (CYP2E1) [7,8,9,10]. It also offers the possibility to study various alcohol and drug interactions [9,38]. However, the hepatic injury does not exceed the stage of fatty liver. Inflammation can only be seen when the dietary fat content is increased [39]. Also, fibrosis cannot be generated. in genetically obese mice fed alcohol [40].

#### 3.3.2. Charles Lieber’s Baboon Model

Since rodents do not resemble human livers with the generation of fibrosis or cirrhosis, it was mandatory to establish a primate model similar to humans. Therefore, isocaloric alcohol-containing diets, similar to those in the rat model, were given to baboons [37]. The baboon liver resembles the human liver quite well. Indeed, baboons develop not only hepatic steatosis but also hepatic lesions beyond the stage of fatty liver such as fibrosis and cirrhosis. Histologically, this hepatic damage is indistinguishable from that of man [37]. Thus, with this animal model, the toxic effect of alcohol to the liver was undoubtedly proven. The model was also helpful in understanding some of the mechanisms by which alcohol damages the liver.

#### 3.3.3. The Tsukamoto-French Intragastric Infusion Model

Alcoholic hepatitis (AH) is a clinical syndrome with high mortality due to liver failure. For this syndrome, an animal model was not available. Drs. Samuel French and Hidekazu Tsukamoto developed a rat model in which ethanol is continuously intragastrically infused [41]. With this technique, blood alcohol concentrations above 200 mg/100 mL blood can be achieved. Therefore, these animals show not only fatty liver, but also severe inflammation similar to AH and fibrosis.

#### 3.3.4. The NIAAA Chronic and Binge Drinking Model

Most recently, a mouse model was introduced which obviously reflects the real situation in man much better compared with the other models, since it consists of chronic ethanol consumption with Lieber-DeCarli diets plus a single binge ethanol feeding [42]. With this model, fatty liver as well as inflammation with neutrophil infiltration can be induced mimicking acute-on-chronic alcoholic liver injury. One advantage of this model is the variation of the fat content of the diet, which offers the possibility to also study the effect of alcohol on NAFLD.

### 3.4. Ethanol Oxidation and Its Consequences on the Liver

Alcoholic liver disease would not exist without hepatic ethanol metabolism. This metabolism includes the oxidation of ethanol to acetaldehyde (AA) by various alcohol dehydrogenases (ADHs) and the microsomal ethanol oxidizing system (MEOS), which is CYP2E1-dependent, as well as by catalase with minor importance. In addition, AA is further oxidized by AA-dehydrogenase (ALDH) to acetate.

#### 3.4.1. Alcohol Dehydrogenase (ADH)

In the sixties and seventies of the last century, it was believed that alcohol metabolism takes place only through the action of ADH. ADH was originally described by Hans Adolf Krebs [43] and it was Jean Pierre von Wartburg who contributed much to the understanding of the action of various ADHs, including the description of an atypical hepatic ADH [44,45,46].

ADH is localized in the cytoplasm of the hepatocytes. ADH requires NAD^+^ as a cofactor, which is reduced to NADH + H^+^ during the metabolism of ethanol to acetaldehyde. With respect to a detailed description of the enzyme, it is referred to review articles [17,47]. Various ADH isozymes exist [17,38,47,48]. Class I ADH (ADH1A, ADH1B, ADH1C), which is the major ADH in the liver, has a Michaelis–Menten constant for ethanol of 0.5–1.0 mM. This equals 0.02–0.05 per mL ethanol. Thus, class I ADH reacts at a relatively low ethanol concentration. Ethanol metabolism via ADH can neither be increased by escalating ethanol concentrations nor after chronic alcohol consumption. ADH 4, which encodes for *π*-ADH, is primarily present in the human liver. ADH 4 30 mM has a much higher Km for ethanol. ADH 5 encodes for χ-ADH present in all tissues with a Km of more than 100 mM. ADH 7 is of special interest since it encodes for σ-ADH, present in the stomach, and is responsible for the first pass metabolism of ethanol [48].

ADH1B and ADH1C show polymorphism. The ADH1B2 allele encodes for an enzyme which is approximately 40 times more active to produce acetaldehyde compared to the ADH1B1 allele. The ADH1C1 allele encodes for an enzyme with 2.5 times more acetaldehyde production compared to the ADH1C2 allele. This plays an important role in cancer development [49,50]. The presence of the ADH1B2 allele is protective for ALD since individuals with this gene show signs of facial flushing associated with tachycardia, sweating, nausea and vomiting due to acetaldehyde. Therefore, these individuals should avoid alcohol completely [51].

Metabolic consequences of the ADH reaction are either due to an increase in hepatic NADH or hepatic acetaldehyde. Production of NADH leads to a change in the hepatic redox potential and has a severe influence on hepatic intermediary metabolism [17,38]. This includes:

1. Increase of fatty acid- and triglyceride synthesis and inhibition of ß-oxidation of fatty acids;
2. Decreased pyruvate and increased lactate concentrations in the liver. This may lead to an inhibition of gluconeogenesis and hypoglycemia. In addition, lactic acidosis with hyperuricemia may occur. Lactate also stimulates hepatic stellate cells (HSCs) to produce collagen;
3. Severe effects on porphyrin metabolism resulting in secondary porphyria;
4. The deleterious effects of acetaldehyde will be discussed below.

Since ethanol metabolism, primarily via ADH, affects hepatic intermediary metabolism, the occurrence of various metabolic diseases, such as hyperhomocysteinemia, porphyria, hyper-uricemia, hypoglycemia, hyperlactacidemia, acidosis, and an altered testosterone/estrogen ratio is favored by chronic ethanol consumption [17,38].

#### 3.4.2. Hepatic Microsomal Ethanol Oxidizing System (MEOS)

It was again Charles Lieber who was the first to describe a non-ADH pathway of ethanol oxidation located in the smooth endoplasmic reticulum of the hepatocyte. He called it the microsomal ethanol oxidizing system (MEOS) [52,53,54,55,56,57]. The MEOS requires molecular oxygen and NADPH as a cofactor. It has an activity optimum of pH 6.9–7.5 and a Michaelis–Menten constant of 7–11 mM for ethanol. The MEOS metabolizes ethanol at higher ethanol concentrations. Major components of the MEOS are CYP2E1 and NADPH, cytochrome c reductase as well as phospholipids [55]. The MEOS is localized in the smooth endoplasmic reticulum of the hepatocyte, which proliferates following chronic alcohol consumption associated with an increase in MEOS activity and CYP2E1. The increased, ethanol metabolism is associated with an elevated generation of acetaldehyde and reactive oxygen species (ROS) [58,59,60]. This increased oxidative stress is of special importance as a pathogenetic mechanism of ALD [61,62,63].

This MEOS pathway was a matter of intensive debate in the nineteen sixties and seventies but was finally accepted as an important mechanism to explain ALD, at least in part.

An experiment with volunteers demonstrated that 40 g of ethanol per day resulted in a significant induction of CYP2E1 after only one week, with a huge interindividual range [64]. CYP2E1 induction may explain an enhanced ethanol metabolism following chronic alcohol ingestion. CYP2E1 activity needs NADPH and reutilizes reducing equivalents from the ADH reaction as NADPH from NADH. The metabolic and clinical consequences of methanol metabolism via the MEOS are as follows [17,38]:

1. Production of hydroxy-ethyl radical, superoxide anion, and hydroxy peroxide, which contribute to liver damage [59,65,66];
2. Interaction of the microsomal ethanol metabolism with the metabolism of a variety of xenobiotics, drugs and carcinogens leading to increased toxicity and carcinogenesis [63];
3. Increased degradation of retinol and retinoic acid, which is relevant in ethanol-mediated carcinogenesis [63,67,68].

#### 3.4.3. First Pass Metabolism (FPM) of Ethanol

For many years, the role of gastric first pass metabolism was unclear. For more than 10 years there was disagreement whether this FPM of ethanol is of gastric or hepatic origin and whether FPM of ethanol plays an important role in the pathogenesis of ALD. The so-called gastric first pass metabolism of alcohol is primarily due to σ-ADH encoded by ADH7 with a Km of 41 mM. However, also γADH encoded by ADH1C and χ-ADH encoded by ADH5 contribute to gastric alcohol metabolism. Various factors such as gender, age, medication (cimetidine, ranitidine, aspirin), the speed of gastric emptying, as well as the integrity and the cell mass of the gastric mucosa (gastritis, presence of *Helicobacter pylori*) affect gastric ADH and ethanol metabolism [69,70,71]. The importance of gastric first pass metabolism of ethanol was heavily questioned between 1985 and 1995. Finally, its role in overall ethanol metabolism was overestimated and it obviously has little if any importance in the pathogenesis of ALD. Its overall contribution to alcohol metabolism is not more than 5–10% in vivo [69,71].

### 3.5. Mechanisms of Hepatic Toxicity

#### 3.5.1. Acetaldehyde

Acetaldehyde, which is a product of cellular and bacterial ethanol oxidation, is extremely toxic and carcinogenic [49,51]; it binds to proteins, leading to structural and functional alterations (for example of mitochondria and microtubules), and induces the formation of neoantigens (host antigens that have been altered enough to generate an immune response) [72,73]. Yedi Israel, and his group from Toronto, were the first to describe acetaldehyde-adduct formation with adequate immune response [72]. Structural mitochondrial alterations caused by acetaldehyde lead to functional impairment, including decreased ATP generation via the respiratory chain, the production of ROS, and a decrease in acetaldehyde dehydrogenase (ALDH) activity, an enzyme located in mitochondria responsible for the metabolism of acetaldehyde to acetate. Acetaldehyde can also bind to deoxyribonucleic acid (DNA), generating carcinogenic DNA adducts [74,75]. Mikko Salaspuro’s work convincingly demonstrated the genetic [76,77] and bacterial [78,79] background of acetaldehyde generation and its role in ethanol-mediated carcinogenesis.

The production of acetaldehyde leads to:

1. Mitochondrial damage with mega-mitochondria [9,17];
2. Damage of the microtubular system with a possible ballooning of the hepatocytes [9,17];
3. Decrease in glutathione (antioxidative defense system) [9,17];
4. Inhibition of the nuclear repair system [80];
5. A disturbed methyl-transfer with decreased levels of the active methyl donor S-adenosyl-methionine (SAME). As a consequence, membrane damage and hypomethylation of DNA may occur, which may contribute to hepatic carcinogenesis [81];
6. Acetaldehyde-protein adducts resulting in neoantigens with the activation of the immune system and production of antibodies [72,73];
7. Acetaldehyde-DNA adduct formation [74,75];
8. Stimulation of fibrogenesis [9].

#### 3.5.2. Oxidative Stress

With the discovery of the role of CYP2E1 in ethanol oxidation, new pathogenetic mechanisms in ALD were elucidated. Charles Lieber [52,53,54,55,56,57], Samuel French [62], Arthur Cederbaum [82,83,84], Emanuelle Albano [59,66,85], Magnus Ingelman Sundberg [65,86], Xiang Dong Wang [39,67,68,87] and our laboratory [63,88] contributed to the understanding of oxidative stress initiated be CYP2E1. During ethanol oxidation via CYP2E1, various ROS, such as ethoxy radical CH_3_CH_2_O^.^, hydroxyethyl radical CH_3_C(^.^)HOH, acetyl radical CH_3_CHO^.^, superoxide radical HO^.^_2_, hydroxyl peroxide H_2_O_2_, hydroxyl radical HO^.^, alkoxyl radical RO^.^, and peroxyl radical ROO^.^, are generated. ROS can bind to proteins, changing their functional and structural properties, and generates neoantigens. In addition, ROS bind directly to, and damage, DNA, or lead to lipid peroxidation with the generation of lipid peroxidation products such as 4-hydroxynonenal (4-HNE) and malondialdehyde (MDA). 4-HNE can bind to DNA bases and form etheno–DNA adducts, which are highly carcinogenic [60,89,90,91,92]. CYP2E1, with its high rate of NADPH oxidase activity, can stimulate the transport of reduced NADH into mitochondria. As a consequence, an increased electron leakage from the hepatocyte mitochondrial respiratory chain and ROS production may occur. CYP2E1 is upregulated by chronic alcohol consumption of more than 40 g of alcohol per day [64]. The induction of CYP2E1 differs between individuals and depends on dietary factors such as the chain length of dietary triglycerides [93]. In addition, iron is another factor which contributes synergistically with ethanol to ROS formation and to the progression of liver disease. Chronic alcohol consumption increases hepatic iron through an increased absorption from the duodenum mediated by decreased hepcidin concentrations [94,95]. Hepatic iron was found to be predictive of death in alcoholic cirrhosis [96].

The importance of CYP2E1-mediated hepatic injury has been demonstrated in experimental mouse models of ALD. The severity of ALD increased in CYP2E1-overexpressing mice and reduced in CYP2E1-deficient mice [97,98,99]. Clomethiazole, a non-competitive inhibitor of CYP2E1 [88], improves ALD and carcinogenesis in experimental animals [100,101]. A significant positive correlation between hepatic CYP2E1 expression, the level of etheno-DNA adducts, and severity of fibrosis has been found in patients with ALD [102]. In addition, CYP2E1 is also involved in the pathogenesis of hepatic steatosis [82,103,104]. Finally, the acetaldehyde-mediated decrease of glutathione contributes to the reduced activity of the antioxidant defense system, which detoxifies ROS [84]. The nuclear factor erythroid 2-related factor 2 (Nrf2) is upregulated after chronic alcohol intake in an in vitro cellular assay [84]. This is an adaptive response of Nrf2, which regulates the expression of important cytoprotective enzymes.

#### 3.5.3. The Gut–Liver Axis: Inflammation through Kupffer Cell Activation by Endotoxins from the Gut

The gut microbiome has attracted much interest as a contributing factor in ALD [105,106]. The theory of increased uptake of endotoxins from the gut and their transport via the portal vein to the liver as a pathogenetic mechanism for ALD was already raised by Christian Bode a long time ago. He also emphasized that alcohol may change the quality of gut bacteria [107,108,109,110,111]. Intestinal dysbiosis and increased intestinal permeability have a key role in the ALD pathogenesis [112]. In fact, alcohol increases the gut permeability to endotoxin/LPS. ALD is associated with reduced synthesis of long-chain fatty acids that support growth of commensal Lactobacilli and integrity of gut barrier.

This different microbiota together with intestinal damage by ethanol, most likely by acetaldehyde, may then lead to an increased uptake of endotoxins. These endotoxins may then bind to toll-like receptor4 and CD10 receptors on Kupffer cells with the activation of nuclear factor kB (NF-kB) and the release of various proinflammatory cytokines (II-8, II-6, TNF, II-1ß) and chemokines (CC-chemokine ligand 2 (CCL2) and an initiation of inflammation [13]. It was Ronald Thurman and his group who followed this idea and demonstrated convincingly that the reduction of intestinal bacteria due to antibiotics, as well as destruction of Kupffer Cells by gadoliume chloride improve experimental ALD in rodents [113,114,115].

In addition, an increase in adaptive immune responses induced by neoantigens (protein adducts with acetaldehyde and ROS) may further contribute to inflammation [116,117].

MicroRNAs are also found in the circulation. MiRNAs are small, non-coding RNAs which post-transcriptionally regulate their target genes. Interestingly, the expression of specific miRNAs is increased whereas others are decreased in ALD [118]. For example, miRNA-155, a key regulator of inflammation, is increased in the liver and circulation in an animal model as well as in patients with alcoholic hepatitis. Furthermore, the inhibition of miRNA-122 is associated with ALD in animals and chronic ethanol consumption and inhibition of miRNA-122 enhances ALD, while restoration of miRNA-122 improved ALD in animals [119,120,121,122].

### 3.6. Sequence of Liver Injury

#### 3.6.1. Alcoholic Fatty Liver

An early pathophysiological response to chronic alcohol consumption is the accumulation of fat (mainly triglycerides, phospholipids and cholesterol esters) in hepatocytes (hepatic steatosis), which can lead to alcoholic fatty liver. Acetate, the end product of ethanol oxidation, is either rapidly secreted into the circulation or converted to acetyl-CoA, which, in turn, contributes to fatty acid synthesis. However, acetate probably has a minimal direct contribution to fatty acid synthesis. Several mechanisms may explain how alcohol affects hepatic fat metabolism [123,124,125,126,127,128,129,130]:

1. Alcohol metabolism increases the hepatic NADH/NAD+ ratio, which inhibits mitochondrial β-oxidation of fatty acids and stimulates fatty acid synthesis resulting in hepatic steatosis.
2. Alcohol consumption up-regulates the hepatic expression of SREBP1c, a transcription factor that stimulates expression of lipogenic genes, which results in increased fatty acid synthesis.
3. Alcohol, probably via acetaldehyde, inactivates peroxisome proliferator-activated receptor-α (PPARα), a nuclear hormone receptor that up-regulates the expression of many genes involved in free fatty acid transport and oxidation.
4. Alcohol inhibits 5′-AMP-activated protein kinase (AMPK) and subsequently inhibits fatty acid synthesis but promotes fatty acid oxidation via the dysregulation of various enzymes involved in fat metabolism.
5. Alcohol consumption affects fatty acid mobilization and clearance. Alcohol consumption induces lipolysis and the death of adipocytes, which increases fatty acids in the circulation and finally their hepatic uptake.
6. Alcohol consumption can also stimulate the influx of lipids from the intestine to the liver.
7. Alcohol activates and inhibits autophagy. While acute alcohol stimulates autophagy and may prevent fat accumulation, chronic alcohol ingestion inhibits autophagy, thereby reducing lipid clearance.

#### 3.6.2. Alcoholic Steatohepatitis (ASH) and Alcoholic Hepatitis (AH)

The pathophysiology of alcoholic steatohepatitis (ASH) has been discussed above. ASH has a typical morphological feature, including hepatocellular injury (with an increase in serum transaminase activity), ballooning of hepatocytes, the presence of Mallory-Denk bodies, necrosis, lobular inflammation, and neutrophilic granulocytic infiltration. This is accompanied by steatosis and fibrosis [13]. Liver biopsy was proposed for a long time as the “gold standard” to study liver disease. However, in the absence of decompensated liver disease, liver biopsy is suitable to establish the diagnosis of ALD, to assess the stage and prognosis, and to exclude concomitant and/or other causes of damage. In fact, three different guidelines, namely, the American Association for the Study of Liver Disease (AASLD), European Association for the Study of the Liver (EASL) and American College of Gastroenterology (ACG), report that biopsy is not routinely recommended for all suspected ALD patients [131]. Liver biopsy, due to its limitations, is not recommended for all patients with suspected alcoholic liver disease (ALD). However, it is useful for establishing the stage and severity of ALD, in case of aggressive forms or severe steatohepatitis requiring specific therapies, for distinguishing comorbid liver pathology. The procedure is invasive and that is the reason for its association with some potential adverse effects and complications, which may be minor (pain or vagal reactions, transient hypotension) or major, such as visceral perforation, bile peritonitis, or significant bleeding. The typical histological features in patients with ALD include steatosis, hepatocellular damage, and lobular inflammation with polymorphonuclear cells infiltration, with a variable degree of fibrosis and lobular distortion that may progress to cirrhosis which confers a high risk of complications (ascites, variceal bleeding, hepatic encephalopathy, renal failure, and bacterial infections).

While ASH is a histomorphologic diagnosis, alcoholic hepatitis (AH) is a clinical syndrome based on ASH. Differential diagnosis of AH includes drug-induced liver injury, sepsis, and ischemic hepatitis. AH is an acute-on chronic disease during episodes of heavy drinking. Thus, the patient’s history of drinking is an important prerequisite for the diagnosis. AH is associated with liver failure leading to severe jaundice and bleeding (dramatic fall in prothrombin time). AH has a poor prognosis with high mortality [13].

Early attempts have been made to predict an outcome. In 1978, Willis Maddrey introduced a severity score of AH that is simple, but still valid to predict the prognosis of the disease [132]. AH was classified on the basis of the evaluation of the risk of one-month mortality, which can be assessed using a discriminant-function score. The discriminant-function score can be calculated as follows: {4.6 × (prothrombin time of the patient–control prothrombin time (in seconds) + serum bilirubin (in mg/dL)}.

Alcoholic hepatitis was defined as severe (one-month mortality higher than 20–30%) for patients with a discriminant-function score ≥ 32.

Almost at the same time, Hector Orrego presented his Orrego index for prognosis estimation [133]. This index consists of some clinical parameters and some laboratory values also based on liver function, but hemoglobin and alkaline phosphatase were also included.

In the following years, more precise severity scores for predicting the one-month short-term mortality have been recommended such as the modified model for end-stage liver disease (MELD) [134] score Age, bilirubin, INR, creatinine (ABIC) [135], and the Glasgow AH scores [136]. Finally, the Lille score has been established, which takes the response to therapy into consideration [137]. Patients with a Lille score ≥ 0.45 following one week of steroid therapy are considered as non-responders. The complete response has a Lille score ≤ 0.16 (91% survival at one month); partial response has a Lille score between 0.16 and 0.56 (79% survival at one month), and the null response has a Lille score > 0.56 (53% survival at one month).

The Barcelona group also introduced a histological score which demonstrates that bilirubin stasis, infiltration of neutrophils, as well as regeneration are important predictors of prognosis [138].

#### 3.6.3. Fibrosis and Cirrhosis

Extracellular matrix production by activated hepatic stellate cells (HSCs) is the key event in hepatic fibrogenesis. In ALD, pericellular and perisinusoidal fibrosis with a “chicken-wire” appearance is characteristic. Chronic alcohol consumption results in the uptake of endotoxins from the gut, which activates Kupffer cells leading to hepatic inflammation that further activates neighboring Kupffer cells that in turn activate HSCs. In addition, acetaldehyde, lactate, and ROS can stimulate hepatic fibrogenesis. They activate HSCs and stimulate immune cells to produce pro-fibrogenic mediators. Alcohol-mediated inhibition of several anti-fibrotic pathways may further contribute to hepatic fibrosis [139]. When fibrogenesis continues, a cirrhotic liver develops. In cirrhosis, hepatic blood flow is disturbed by a narrowing of vascular structures (including sinusoids) within the hepatic lobule. As a result, portal hypertension with complications such as ascites and esophageal varices may occur. In addition, due to a loss of hepatocytes, liver function deteriorates.

#### 3.6.4. Hepatocellular Cancer

The fact that chronic alcohol consumption may result in HCC via cirrhosis is not new [140]. Indeed, alcoholic beverages are group 1 carcinogens (known to be carcinogenic to humans) per classification by the International Agency for Research on Cancer [141]. Alcohol is a procarcinogen that requires its bioconversion to a primary carcinogenic metabolite, acetaldehyde. Individuals with ALDH2*2,1 heterozygosity (40% of Asians) coding for an ALDH2 with less than 15% of activity, as compared to the normal ALDH mutation, have an increased risk of esophageal cancer when they consume alcohol chronically. This clearly shows the carcinogenicity of acetaldehyde [49,51,76,77]. Acetaldehyde is electrophilic and, as mentioned before, forms an adduct with DNA and inter-strand cross-links. DNA mutations can occur especially when DNA repair mechanisms are insufficient. This inhibition of DNA repair is also due to acetaldehyde. Acetaldehyde inhibits the activity of the DNA repair enzyme O6 -methylguanine DNA methyltransferase, causing both genotoxicity and DNA repair failure [80].

As mentioned above, ROS produced by alcohol-associated CYP2E1 induction generates lipid peroxidation products such as 4-hydroxy-nonenal (4-HNE) and malondialdehyde (MDA). MDA increases acetaldehyde-adduct formation significantly, resulting in the formation of a highly reactive, hybrid MDA–acetaldehyde adduct [142]. While acetaldehyde protein-adducts and acetaldehyde DNA adducts cause the generation of neoantigens and mutations, reduced glutathione amplify oxidant stress and cytotoxicity (see above). In addition, lipid peroxidation products, such as 4-HNE and MDA, bind to DNA, forming highly carcinogenic etheno DNA adducts [60]. In summary, oxidative stress, either generated through alcohol-induced inflammation (alcoholic hepatitis) or via induced CYP2E1, causes hepatocellular DNA damage and contributes to cancer development.

It has also been shown, in early studies by the Lieber group, that induced CYP2E1 converts other procarcinogens to active carcinogens, including nitrosamines [143].

Epigenetic changes induced by severe chronic alcohol consumption can lead to chromosomal instability [144]. Hypomethylation of promoters for oncogenes causes a disturbed activation and a loss of a normal expression pattern. Hypermethylation of promoters of genes involved in cellular differentiation or DNA repair promotes transformation [145].

Finally, activated stellate cells (by macrophages) not only promote fibrosis, but also HCC formation via the production of matrix and soluble factors that support tumor cell survival and growth [146], as well as by tumor-initiating stem cell-like cells mediated hepatocarcinogenesis induced by hepatotoxins and carcinogens [13].

### 3.7. Clinical Aspects of ALD

#### 3.7.1. Diagnosis

Various laboratory tests are specific for chronic alcohol consumption, including the mean corpuscular volume of the erythrocytes (MCV), serum gamma-glutamyl-transferase (GGT) activity, and serum uric acid [17]. In addition, more specific markers are carbohydrate-deficient transferrin (CDT) [147,148], phosphatidyl-ethanol (PtE) [149,150], and ethyl-glucuronide (EtG) in the urine [151]. Since serum GGT activity is elevated in any type of liver disease, it loses its specificity for alcohol in the presence of any liver disease [17,148,152].

When hepatocytes are destroyed as a cause of alcohol toxicity, the activity of serum aspartate-amino transaminase (AST) is found to be higher than that of alanine-amino transaminase (DeRitis ratio) [17].

Alcoholic fatty liver can be diagnosed by ultrasound. However, this method is not very accurate. More recently it was reported that the controlled attenuation parameter (CAP) represents an excellent method to quantify hepatic steatosis [153].

Hepatic inflammation is associated with increased serum transaminase activities (see above). Alcohol-mediated inflammation leads to the release of pro-inflammatory cytokines into the circulation. Non-invasive biomarkers such as cytokeratins CK 8 (M-30) and CK 18 (M-65) are employed to measure cell death by apoptosis and necrosis in blood samples [154].

For hepatic fibrosis, a battery of serum markers has been introduced over the years. This includes, among others, hyaluronic acid (HA) and procollagen III N-terminal propeptide (PIIINP), type VI and type XIV collagens, prothrombin, chitinase 3-like protein 1 (CHI3L1), and an enhanced liver fibrosis panel consisting of HA, PIIINP, and tissue inhibitor of metalloproteinases (TIMP1) [13]. Most of these markers do not have a convincing sensitivity and specificity for alcoholic fibrosis, except perhaps hyaluronic acid [155,156]. With the introduction of transient elastography (Fibroscan), the diagnostic accuracy of fibrosis has increased significantly. This method is a tool to determine at least F3 and F4 fibrosis and is also valuable for controlling the course of the disease over time [157,158].

In advanced liver disease, hepatic function deteriorates. This is best reflected by the determination of serum albumin levels, by measurement of blood coagulation and serum bilirubin concentrations.

With the introduction of transient elastography, liver biopsy has lost its importance in the follow-up. However, when alcohol as a cause of the hepatic disease is questionable or when various causes in combination with alcohol may affect the liver, such as hepatitis B or C, non-alcoholic fatty liver disease (NAFLD) and hepatic histology may be helpful.

#### 3.7.2. Prognosis

In all stages of ALD, abstinence improves survival significantly, even at the stage of cirrhosis [159]. Under alcohol abstinence, fatty liver normalizes and moderate hepatic fibrosis may regress. In compensated and even decompensated cirrhosis of the liver, mortality is significantly reduced when alcohol intake is stopped as compared to continuous drinking. Various studies with compensated cirrhosis have shown a five-year survival of 50 to 80% with abstinence, as compared to 40 to 68% with continuous drinking [160,161,162,163,164,165,166,167,168]. Thus, alcohol abstinence is a good therapy and the success of each new therapy has to be compared to abstinence.

#### 3.7.3. Treatment

##### Abstinence

The fundamental basis of therapy is abstinence. We have shown recently that all types and severities of ALD improve with abstinence [159] (see prognosis). Patients with alcohol use disorders need professional help by a psychiatrist involving talking therapy alone or in groups, eventually accompanied by anti-graving drugs [169].

##### Medication for AH

Fifty years ago, Willis Maddrey introduced steroids for the therapy of AH [132]. Since then, no fundamental new therapy has been developed. As in 50 years ago, steroid therapy is still recommended if the patient responds [138,170]. Since many of the patients are malnourished, hyperalimentation is another important therapeutic option [171,172]. Additionally, the administration of N-acetyl cysteine has shown a significant beneficial effect on survival [173].

##### Treatment of Cirrhosis

Specific treatment for alcoholic cirrhosis still does not exist. Therapy of complications of cirrhosis includes therapy of ascites, encephalopathy, portal hypertension, and HCC (see guidelines for therapy of liver and HCC; [174,175]).

##### Liver Transplantation

In 1988, Thomas Starzl was the first who performed a liver transplantation (LT) for end-stage ALD [176]. As expected, this topic was controversially debated then, and still is today. Despite the fact that the survival of patients following LT for ALD was comparable to or even better as survival for patients with other liver diseases, LT for ALD was taken critically by the public as well as by the medical profession. It was questioned whether it is acceptable to give a liver to somebody who damaged his/her own liver by drinking (a self-induced disease), especially when organs are rare. This opinion was further enhanced when the football star Georg Best continued drinking following liver transplantation and finally died due to cirrhosis in the new transplanted liver.

However, as time changes, opinions do to. It is a fact that alcohol use disorders have a genetic background of approximately 60% [177]. Since alcohol dependency is difficult to treat, with a high relapse rate, despite psychiatric therapy sometimes flanked by medication, the relapse rate is high. To minimize relapse the rate following LT, the so-called “six-month rule” was introduced. However, the “six-month rule” is based on an observation of 11 patients [178]. For further information, refer to a recent editorial [179]. In summary, pre-transplantation abstinence (six months or more) is a good inclusion criterion (i.e., 88% sobriety post-transplant) but a poor exclusion criterion (i.e., with less than six months abstinence only 41% returned to drinking) [179,180]. In general, the relapse rate after LT for ALD is low, with 10 to 15%, depending on the definition.

Mathurin and colleagues demonstrated, in a landmark study, that patients with AH who were not abstinent before LT had a surprisingly low relapse within the first two years [181]. Thus, the “six-month rule” is not the last wisdom. It is now well-established that an expert psycho-social evaluation is of major importance and the integration of a psychiatrist experienced in the diagnosis and treatment of alcoholism is mandatory [182].

## 4. Summary and Conclusions

It took over 70 years to recognize ALD as a disease on its own with certain risk factors including genetics. This review describes the long way from clinical observations to the understanding of the biochemical and molecular basis of ALD as we see it today [183,184]. It was the purpose of this review to describe pioneering work and landmark contributions during 70 years of alcohol research.

One reason for this slow development was that little public money was available to support alcohol research, and that the alcohol-producing industry has always tried to prevent alcohol from being seen by the public as a danger. Since alcohol consumption is a major health problem, and since the liver is the predominant target of alcohol, resulting in ALD with a high number of deaths, disabilities, and costs for the public, an action plan to fight alcohol consumption and ALD is urgently needed. This includes translational research activities to gain a better understanding of the pathophysiology of ALD, especially of AH, with the urgent need of more effective therapy. On the basis of the published data, it is possible to support the hypothesis that the combination of effective anti-inflammatory therapies and liver support could improve survival for ALD patients.

This plan may also include training programs for general practitioners to identify individuals with alcohol problems early on, and to transfer them to a psychiatrist and/or hepatologist. Finally, preventive actions to raise awareness of the dangers associated with alcohol consumption is necessary. This may include an increased taxation of alcoholic beverages, a reduced availability of alcoholic beverages, or a complete ban of alcohol advertisements.

## Data Availability

The data presented in this study are openly available in all the cited articles.

## Abbreviations

AA	Acetaldehyde.
AASLD	American Association for the Study of the Liver Disease
ABIC	Age, bilirubin, INR, creatinine
ACG	American College of Gastroenterology
ADH	alcohol dehydrogenase
ALD	alcoholic liver disease
ALDH	acetaldehyde dehydrogenase
CH_3_CHO	acetyl radical^.^
CH_3_CH_2_O	ethoxy radical^.^
CYP2E1	cytochrome P450 2E1
DNA	deoxyribonucleic acid
FPM	first pass metabolism
HCC	hepatocellular carcinoma
HO	hydroxyl radical^.^
HO^.^_2_	superoxide radical
H_2_O_2_ MELD	hydroxyl peroxide modified model for end-stage liver disease
MEOS	microsomal ethanol oxidizing system
ROS	reactive oxygen species,
4-HNE	4-hydroxynonenal
LT	liver transplantation
MDA	malondialdehyde
RO	alkoxyl radical^.^
ROO	peroxyl radical
SAME	S-adenosyl-methionine
